# 2,2-Bis(hy­droxy­meth­yl)-2,3-dihydro-1*H*-pyrrolizin-1-one

**DOI:** 10.1107/S1600536810018970

**Published:** 2010-06-05

**Authors:** Yousuf Ali, Yu Peng, Erbing Hua, Nighat Afza, Rashid Ali Khan

**Affiliations:** a Department of Pharmaceutical Engineering, Biotechnology College, Tianjin University of Science & Technology (TUST), Tianjin 300457, People’s Republic of China; b Pharmaceutical Research Center, PCSIR Laboratories Complex, Karachi 75280, Pakistan

## Abstract

The title compound, C_9_H_11_NO_3_, was prepared by an Aldol reaction of 2,3-dihydro-1*H*-pyrrolizin-1-one with formaldehyde. The asymmetric unit contains six mol­ecules. The pyrrolizine ring system in each mol­ecule is planar, the maximum atomic deviation being 0.066 (2) Å. In the crystal structure, mol­ecules are liked together by an extensive O—H⋯O hydrogen-bonding network.

## Related literature

For general background to 2,3-dihydro­pyrrolizine derivatives and for the biological activity of related compounds, see: Meinwald & Meinwald (1965 ▶); Skvortsov & Astakhova (1992 ▶); Albrecht *et al.* (2008 ▶); Mishra *et al.* (2008 ▶); Morúaa *et al.* (2009 ▶). For the preparation of the starting material, see: Clemo & Ramage (1931 ▶); Braunholtz *et al.* (1962 ▶). For a related structure, see: Ali *et al.* (2010 ▶).
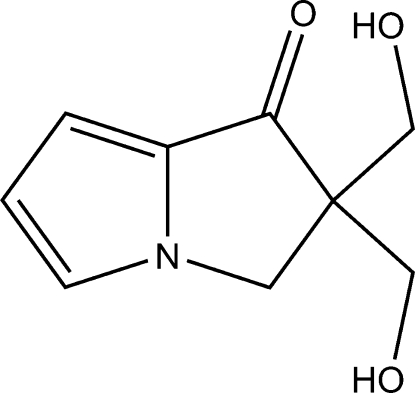


## Experimental

### 

#### Crystal data

C_9_H_11_NO_3_
*M* *_r_* = 181.19
Monoclinic, 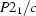

*a* = 10.2601 (8) Å
*b* = 39.863 (3) Å
*c* = 12.412 (1) Å
β = 90.273 (6)°
*V* = 5076.4 (7) Å^3^
*Z* = 24
Cu *K*α radiation
μ = 0.90 mm^−1^
*T* = 113 K
0.22 × 0.18 × 0.16 mm

#### Data collection

Rigaku Saturn70 CCD diffractometer
Absorption correction: multi-scan (*CrystalClear*; Rigaku, 2009 ▶) *T*_min_ = 0.827, *T*_max_ = 0.870
51559 measured reflections
9889 independent reflections
8550 reflections with *I* > 2σ(*I*)
*R*_int_ = 0.059

#### Refinement

*R*[*F* ^2^ > 2σ(*F* ^2^)] = 0.045
*wR*(*F* ^2^) = 0.122
*S* = 1.09
9889 reflections
751 parameters
H atoms treated by a mixture of independent and constrained refinement
Δρ_max_ = 0.26 e Å^−3^
Δρ_min_ = −0.27 e Å^−3^

### 

Data collection: *CrystalClear* (Rigaku, 2009 ▶); cell refinement: *CrystalClear*; data reduction: *CrystalClear*; program(s) used to solve structure: *SHELXS97* (Sheldrick, 2008 ▶); program(s) used to refine structure: *SHELXL97* (Sheldrick, 2008 ▶); molecular graphics: *ORTEP-3* (Farrugia, 1997 ▶); software used to prepare material for publication: *publCIF* (Westrip, 2010 ▶).

## Supplementary Material

Crystal structure: contains datablocks I, global. DOI: 10.1107/S1600536810018970/xu2763sup1.cif

Structure factors: contains datablocks I. DOI: 10.1107/S1600536810018970/xu2763Isup2.hkl

Additional supplementary materials:  crystallographic information; 3D view; checkCIF report

## Figures and Tables

**Table 1:** Hydrogen-bond geometry (Å, °)

*D*—H⋯*A*	*D*—H	H⋯*A*	*D*⋯*A*	*D*—H⋯*A*
O2—H2⋯O3^i^	0.91 (3)	1.85 (3)	2.7513 (19)	172 (2)
O3—H3⋯O4	0.87 (3)	1.95 (3)	2.7612 (17)	154 (3)
O5—H5⋯O6^ii^	0.82 (3)	1.98 (3)	2.7776 (17)	166 (2)
O6—H6⋯O1^iii^	0.90 (3)	1.80 (3)	2.6801 (16)	168 (2)
O8—H8⋯O16	0.88 (3)	1.85 (3)	2.7219 (16)	168 (2)
O9—H9⋯O11^iv^	0.84 (3)	1.96 (3)	2.7841 (18)	168 (2)
O11—H11⋯O13^v^	0.85 (2)	1.92 (2)	2.7239 (16)	157 (2)
O12—H12⋯O8	0.84 (3)	2.00 (3)	2.8186 (18)	167 (2)
O14—H14⋯O18	0.87 (3)	1.95 (3)	2.8063 (18)	168 (2)
O15—H15⋯O10^vi^	0.93 (3)	1.86 (3)	2.7290 (16)	155 (3)
O17—H17⋯O15^v^	0.88 (3)	1.96 (3)	2.8144 (18)	166 (3)
O18—H18⋯O7^vii^	0.86 (2)	1.92 (2)	2.7579 (17)	168 (2)

Symmetry codes: (i) 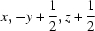; (ii) 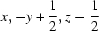; (iii) 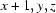; (iv) 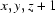; (v) 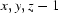; (vi) 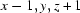; (vii) 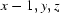.

**Fig. 1.**
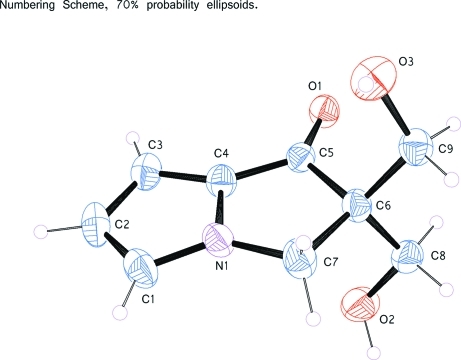
View of the single molecule showing atom numbering scheme at 70% ellipsoids probability level. Hydrogen atoms are drawn as spheres of an arbitrary radius.

**Fig. 2.**
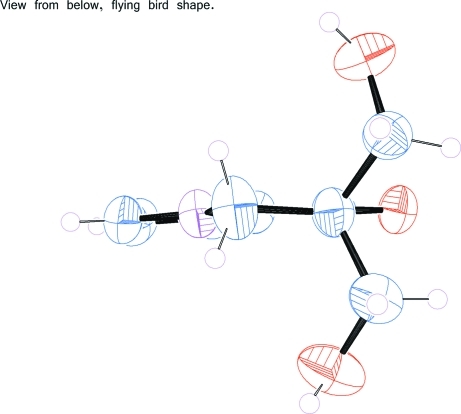
View of molecule from below, showing two hydroxyl bearign methylene groups, one above and one below the ring system.

**Fig. 3.**
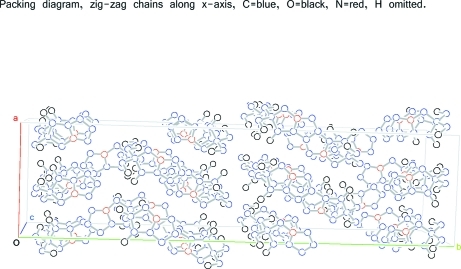
Packing diagram of cell unit showing discrete zigzag chains.

**Fig. 4.**
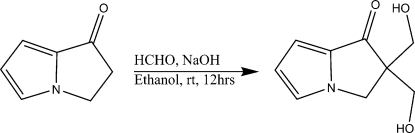
Chemical Reaction Scheme.

## supplementary crystallographic information

### Comment

Title compound was prepared in order to synthesize new derivatives of this series. Derivatives of 2,3-dihydropyrrolizine became known through studies of their synthesis (Clemo & Ramage, 1931; Braunholtz *et al.*, 1962), and isolation from natural source (Meinwald & Meinwald, 1965). Depending on their structure derivatives of 2,3-dihydropyrrolizine have shown merit as analgesics, anti-inflammatory agents, myorelaxants, inhibitors of thrombocyte aggregation, fibrinolytics, temperature-lowering substances and drugs for the treatment of glaucoma and conjunctivitis (Skvortsov *et al.*, 1992). The most important of these, Ketorolac, is reported in literature as one of the most effective nonsteroidal anti-inflammatory drugs to alleviate renoureteral colic (Morúaa *et al.*, 2009). But it suffers from the general side effects of NSAIDs, owing to presence of free carboxylic acid group (Mishra *et al.*, 2008). Licofelone(2-[6-(4-Chlorophenyl)-2,2- dimethyl-7-phenyl-2,3-dihydro-1Hpyrrolizin-5-yl] acetic acid) is a dual inhibitor of both cyclooxygenase isoforms and 5-lipoxygenase (Albrecht *et al.*, 2008). Crystal structures of related molecules are reported (Ali *et al.*, 2010).

Numbering scheme for the title compound is shown in an ORTEP (Farrugia, 1997) plot of the molecule at 70% ellipsoid probability limit (Fig. 1). In the crystal structure of the title compound, molecules are connected via intermolecular O—H···O hydrogen bonds (Table 1) to form discrete zigzag chains (Fig. 3). Assymetric unit contains, nearly identical, six molecules. The pyrrolizin ring system is almost planner [maximum deviation = 0.066 (2) Å] with two methylene groups, bearing hydroxyl groups, above and below the plane of the ring. Thus forming a flying bird like shape (Fig. 2).

### Experimental

A 100 ml round bottom one neck flask, equipped with a magnetic stirrer, is charged with 20 ml of Ethanol 95%, 4.03 g (33.3 mmol) of 2,3-dihydro-1*H*-pyrrolizin-1-one and 8 ml formalin (formaldehyde 37% aquous). The reaction vessel is immersed in a bath of cold water and l.6 ml of 5% sodium hydroxide aqueous solution is added slowly (during about 10 minutes) from a dropping funnel. The rate of addition was adjusted so that the temperature remains between 293 and 298 K. The mixture was stirred overnight at room temperature. Dilute hydrochloric acid was added to render the mixture just acidic to litmus paper (pH = 5 to 6). Solvents were removed under diminished pressure. The residue was subjected directly to Flash Column chromatography (Petroleum Ether : Ethyl Acetate = 1:1) to give 3.77 g (62.5%) of crystalline mass, m.p. 121-122 °C. Single crystals for X-Ray analysis were prepared by evaporation from a methanol solution.

### Refinement

Hydroxy H atoms were located in a difference Fourier map and refined isotropically. Other H atoms were geometrically positioned and refined using a riding model with U_iso_(H) = 1.2U_eq_(C) and with C—H = 0.95 or 0.99 Å.

### Crystal data

**Table:**

C_9_H_11_NO_3_	*F*(000) = 2304
*M**_r_* = 181.19	*D*_x_ = 1.422 Mg m^−^^3^
Monoclinic, *P*2_1_/*c*	Melting point: 395 K
Hall symbol: -P 2ybc	Cu *K*α radiation, λ = 1.54187 Å
*a* = 10.2601 (8) Å	Cell parameters from 5462 reflections
*b* = 39.863 (3) Å	θ = 27.5–72.2°
*c* = 12.412 (1) Å	µ = 0.90 mm^−^^1^
β = 90.273 (6)°	*T* = 113 K
*V* = 5076.4 (7) Å^3^	Prism, colourless
*Z* = 24	0.22 × 0.18 × 0.16 mm

### Data collection

**Table:**

Rigaku Saturn70 CCD camera diffractometer	9889 independent reflections
Radiation source: fine-focus sealed tube	8550 reflections with *I* > 2σ(*I*)
multilayer	*R*_int_ = 0.059
ω scans	θ_max_ = 72.7°, θ_min_ = 2.2°
Absorption correction: multi-scan (*CrystalClear*; Rigaku, 2009)	*h* = −12→12
*T*_min_ = 0.827, *T*_max_ = 0.870	*k* = −48→48
51559 measured reflections	*l* = −12→14

### Refinement

**Table:**

Refinement on *F*^2^	Primary atom site location: structure-invariant direct methods
Least-squares matrix: full	Secondary atom site location: difference Fourier map
*R*[*F*^2^ > 2σ(*F*^2^)] = 0.045	Hydrogen site location: inferred from neighbouring sites
*wR*(*F*^2^) = 0.122	H atoms treated by a mixture of independent and constrained refinement
*S* = 1.09	*w* = 1/[σ^2^(*F*_o_^2^) + (0.0583*P*)^2^ + 1.4861*P*] where *P* = (*F*_o_^2^ + 2*F*_c_^2^)/3
9889 reflections	(Δ/σ)_max_ < 0.001
751 parameters	Δρ_max_ = 0.26 e Å^−^^3^
0 restraints	Δρ_min_ = −0.27 e Å^−^^3^

### Special details

**Table:**

Geometry. All esds (except the esd in the dihedral angle between two l.s. planes) are estimated using the full covariance matrix. The cell esds are taken into account individually in the estimation of esds in distances, angles and torsion angles; correlations between esds in cell parameters are only used when they are defined by crystal symmetry. An approximate (isotropic) treatment of cell esds is used for estimating esds involving l.s. planes.
Refinement. Refinement of *F*^2^ against ALL reflections. The weighted *R*-factor wR and goodness of fit *S* are based on *F*^2^, conventional *R*-factors *R* are based on *F*, with *F* set to zero for negative *F*^2^. The threshold expression of *F*^2^ > σ(*F*^2^) is used only for calculating *R*-factors(gt) etc. and is not relevant to the choice of reflections for refinement. *R*-factors based on *F*^2^ are statistically about twice as large as those based on *F*, and *R*- factors based on ALL data will be even larger.

### Fractional atomic coordinates and isotropic or equivalent isotropic displacement parameters (Å^2^)

**Table:**

	*x*	*y*	*z*	*U*_iso_*/*U*_eq_	
O1	0.00788 (10)	0.23601 (3)	0.79919 (9)	0.0209 (2)	
O2	0.12194 (13)	0.26396 (3)	1.04690 (11)	0.0315 (3)	
H2	0.175 (2)	0.2699 (6)	1.102 (2)	0.050 (7)*	
O3	0.29990 (12)	0.22299 (3)	0.70802 (10)	0.0285 (3)	
H3	0.376 (3)	0.2300 (7)	0.688 (2)	0.062 (8)*	
O4	0.50703 (11)	0.24375 (3)	0.58345 (9)	0.0224 (3)	
O5	0.60266 (12)	0.26017 (3)	0.32413 (10)	0.0245 (3)	
H5	0.651 (2)	0.2602 (6)	0.272 (2)	0.042 (7)*	
O6	0.79773 (11)	0.23639 (3)	0.67012 (10)	0.0240 (3)	
H6	0.874 (3)	0.2378 (6)	0.706 (2)	0.044 (7)*	
O7	1.09436 (11)	0.06150 (3)	0.47694 (9)	0.0240 (3)	
O8	0.80230 (11)	0.07468 (3)	0.38956 (9)	0.0230 (3)	
H8	0.728 (2)	0.0737 (6)	0.354 (2)	0.045 (7)*	
O9	0.99084 (12)	0.03716 (3)	0.72620 (11)	0.0273 (3)	
H9	0.941 (2)	0.0409 (6)	0.778 (2)	0.041 (7)*	
O10	1.09012 (11)	0.05039 (3)	−0.03329 (10)	0.0229 (3)	
O11	0.79658 (12)	0.04535 (3)	−0.12018 (9)	0.0248 (3)	
H11	0.722 (2)	0.0404 (6)	−0.1456 (19)	0.037 (6)*	
O12	0.98992 (12)	0.06862 (3)	0.22557 (10)	0.0250 (3)	
H12	0.943 (2)	0.0722 (6)	0.279 (2)	0.044 (7)*	
O13	0.59009 (10)	0.03485 (3)	0.74524 (9)	0.0209 (2)	
O14	0.47801 (12)	0.05834 (3)	0.49770 (10)	0.0278 (3)	
H14	0.428 (2)	0.0666 (6)	0.447 (2)	0.041 (6)*	
O15	0.29842 (12)	0.03026 (3)	0.84705 (10)	0.0277 (3)	
H15	0.217 (3)	0.0385 (7)	0.868 (2)	0.065 (8)*	
O16	0.59239 (10)	0.07059 (3)	0.25528 (9)	0.0220 (2)	
O17	0.49233 (12)	0.04752 (3)	−0.00169 (10)	0.0255 (3)	
H17	0.438 (3)	0.0448 (7)	−0.056 (2)	0.054 (8)*	
O18	0.29584 (11)	0.07697 (3)	0.33930 (10)	0.0219 (2)	
H18	0.226 (2)	0.0734 (6)	0.3752 (19)	0.036 (6)*	
N1	0.21112 (13)	0.30568 (3)	0.82425 (11)	0.0209 (3)	
N2	0.70994 (13)	0.18680 (3)	0.45337 (11)	0.0189 (3)	
N3	0.88997 (12)	0.11537 (3)	0.61882 (11)	0.0185 (3)	
N4	0.87478 (12)	0.11657 (3)	0.00760 (11)	0.0188 (3)	
N5	0.38885 (13)	0.10504 (3)	0.72801 (11)	0.0188 (3)	
N6	0.37989 (12)	0.12235 (3)	0.11227 (11)	0.0188 (3)	
C1	0.21244 (17)	0.33884 (4)	0.80508 (15)	0.0252 (4)	
H1	0.2813	0.3539	0.8218	0.030*	
C2	0.09387 (17)	0.34708 (4)	0.75598 (15)	0.0270 (4)	
H2A	0.0676	0.3689	0.7339	0.032*	
C3	0.02071 (16)	0.31774 (4)	0.74498 (14)	0.0233 (3)	
H3A	−0.0636	0.3158	0.7136	0.028*	
C4	0.09541 (15)	0.29189 (4)	0.78898 (13)	0.0196 (3)	
C5	0.09389 (15)	0.25707 (4)	0.81614 (13)	0.0174 (3)	
C6	0.22115 (15)	0.24870 (4)	0.87559 (13)	0.0189 (3)	
C7	0.30154 (16)	0.28135 (4)	0.87220 (16)	0.0255 (4)	
H7A	0.3284	0.2883	0.9455	0.031*	
H7B	0.3802	0.2786	0.8271	0.031*	
C8	0.18828 (16)	0.23812 (4)	0.99139 (14)	0.0228 (3)	
H8A	0.1331	0.2178	0.9896	0.027*	
H8B	0.2698	0.2325	1.0305	0.027*	
C9	0.28809 (16)	0.21894 (4)	0.82123 (14)	0.0214 (3)	
H9A	0.3761	0.2160	0.8530	0.026*	
H9B	0.2376	0.1983	0.8361	0.026*	
C10	0.71767 (17)	0.15398 (4)	0.42930 (14)	0.0247 (4)	
H10	0.7880	0.1431	0.3941	0.030*	
C11	0.60312 (18)	0.13877 (4)	0.46555 (15)	0.0281 (4)	
H11A	0.5812	0.1157	0.4584	0.034*	
C12	0.52670 (17)	0.16340 (4)	0.51397 (15)	0.0250 (4)	
H12A	0.4438	0.1603	0.5462	0.030*	
C13	0.59529 (15)	0.19350 (4)	0.50604 (13)	0.0185 (3)	
C14	0.59120 (14)	0.22819 (4)	0.53385 (13)	0.0176 (3)	
C15	0.71635 (15)	0.24481 (4)	0.49083 (13)	0.0175 (3)	
C16	0.79390 (15)	0.21608 (4)	0.43832 (14)	0.0200 (3)	
H16A	0.8086	0.2205	0.3609	0.024*	
H16B	0.8791	0.2129	0.4746	0.024*	
C17	0.67828 (15)	0.27266 (4)	0.41111 (13)	0.0199 (3)	
H17A	0.6280	0.2901	0.4495	0.024*	
H17B	0.7583	0.2832	0.3826	0.024*	
C18	0.78953 (15)	0.26041 (4)	0.58545 (13)	0.0202 (3)	
H18A	0.8781	0.2671	0.5628	0.024*	
H18B	0.7430	0.2807	0.6108	0.024*	
C19	0.88204 (17)	0.14731 (4)	0.65250 (15)	0.0250 (4)	
H19	0.8126	0.1570	0.6922	0.030*	
C20	0.99474 (18)	0.16368 (4)	0.61835 (16)	0.0289 (4)	
H20	1.0159	0.1865	0.6311	0.035*	
C21	1.07093 (17)	0.14066 (4)	0.56230 (15)	0.0256 (4)	
H21	1.1528	0.1449	0.5296	0.031*	
C22	1.00406 (15)	0.11023 (4)	0.56347 (13)	0.0198 (3)	
C23	1.00883 (15)	0.07613 (4)	0.52823 (12)	0.0180 (3)	
C24	0.88236 (14)	0.05865 (4)	0.56444 (13)	0.0172 (3)	
C25	0.80686 (15)	0.08571 (4)	0.62717 (13)	0.0188 (3)	
H25A	0.7203	0.0898	0.5941	0.023*	
H25B	0.7950	0.0791	0.7033	0.023*	
C26	0.80900 (16)	0.04725 (4)	0.46370 (14)	0.0219 (3)	
H26A	0.8548	0.0281	0.4302	0.026*	
H26B	0.7200	0.0399	0.4831	0.026*	
C27	0.91564 (16)	0.02804 (4)	0.63456 (14)	0.0231 (3)	
H27A	0.8339	0.0172	0.6584	0.028*	
H27B	0.9649	0.0116	0.5911	0.028*	
C28	0.85751 (17)	0.14907 (4)	−0.01890 (14)	0.0244 (4)	
H28	0.7856	0.1628	0.0006	0.029*	
C29	0.96452 (18)	0.15888 (4)	−0.08015 (15)	0.0290 (4)	
H29	0.9785	0.1805	−0.1098	0.035*	
C30	1.04757 (17)	0.13129 (4)	−0.09025 (14)	0.0254 (4)	
H30	1.1281	0.1307	−0.1277	0.031*	
C31	0.98977 (15)	0.10492 (4)	−0.03510 (13)	0.0193 (3)	
C32	1.00243 (14)	0.07002 (4)	−0.00908 (12)	0.0170 (3)	
C33	0.88121 (14)	0.05919 (4)	0.05423 (13)	0.0168 (3)	
C34	0.79884 (15)	0.09140 (4)	0.06617 (13)	0.0198 (3)	
H34A	0.7113	0.0885	0.0336	0.024*	
H34B	0.7891	0.0977	0.1429	0.024*	
C35	0.81048 (15)	0.03267 (4)	−0.01264 (13)	0.0195 (3)	
H35A	0.8612	0.0115	−0.0131	0.023*	
H35B	0.7237	0.0280	0.0185	0.023*	
C36	0.92196 (16)	0.04439 (4)	0.16262 (13)	0.0207 (3)	
H36A	0.8436	0.0368	0.2020	0.025*	
H36B	0.9787	0.0247	0.1507	0.025*	
C37	0.38558 (17)	0.13759 (4)	0.75589 (14)	0.0246 (4)	
H37	0.3169	0.1529	0.7414	0.030*	
C38	0.50179 (18)	0.14466 (4)	0.81014 (15)	0.0286 (4)	
H38	0.5260	0.1658	0.8392	0.034*	
C39	0.57621 (16)	0.11548 (4)	0.81455 (14)	0.0236 (4)	
H39	0.6597	0.1129	0.8470	0.028*	
C40	0.50416 (15)	0.09073 (4)	0.76208 (13)	0.0182 (3)	
C41	0.50526 (14)	0.05629 (4)	0.72993 (12)	0.0160 (3)	
C42	0.37586 (14)	0.04861 (4)	0.67170 (13)	0.0163 (3)	
C43	0.30152 (15)	0.08220 (4)	0.66999 (14)	0.0211 (3)	
H43A	0.2864	0.0900	0.5952	0.025*	
H43B	0.2166	0.0802	0.7070	0.025*	
C44	0.40411 (15)	0.03513 (4)	0.55893 (13)	0.0197 (3)	
H44A	0.4527	0.0138	0.5649	0.024*	
H44B	0.3208	0.0305	0.5212	0.024*	
C45	0.30486 (15)	0.02149 (4)	0.73617 (13)	0.0195 (3)	
H45A	0.2155	0.0186	0.7072	0.023*	
H45B	0.3513	−0.0001	0.7284	0.023*	
C46	0.36546 (17)	0.15442 (4)	0.08148 (14)	0.0242 (4)	
H46	0.2952	0.1633	0.0403	0.029*	
C47	0.47214 (17)	0.17239 (4)	0.12078 (15)	0.0274 (4)	
H47	0.4874	0.1957	0.1112	0.033*	
C48	0.55255 (17)	0.15008 (4)	0.17670 (14)	0.0247 (4)	
H48	0.6324	0.1553	0.2119	0.030*	
C49	0.49328 (15)	0.11869 (4)	0.17092 (13)	0.0187 (3)	
C50	0.50457 (14)	0.08431 (4)	0.20370 (12)	0.0168 (3)	
C51	0.38331 (14)	0.06528 (4)	0.16315 (13)	0.0168 (3)	
C52	0.30237 (15)	0.09173 (4)	0.10148 (13)	0.0196 (3)	
H52A	0.2916	0.0854	0.0248	0.024*	
H52B	0.2152	0.0945	0.1342	0.024*	
C53	0.42590 (16)	0.03599 (4)	0.09137 (13)	0.0204 (3)	
H53A	0.4839	0.0209	0.1330	0.024*	
H53B	0.3482	0.0229	0.0691	0.024*	
C54	0.31093 (15)	0.05104 (4)	0.26027 (13)	0.0195 (3)	
H54A	0.2243	0.0426	0.2375	0.023*	
H54B	0.3607	0.0321	0.2915	0.023*	

### Atomic displacement parameters (Å^2^)

**Table:**

	*U*^11^	*U*^22^	*U*^33^	*U*^12^	*U*^13^	*U*^23^
O1	0.0194 (5)	0.0193 (6)	0.0238 (6)	−0.0030 (4)	−0.0024 (4)	0.0027 (5)
O2	0.0339 (7)	0.0348 (7)	0.0258 (7)	0.0121 (5)	−0.0037 (6)	−0.0075 (6)
O3	0.0235 (6)	0.0382 (7)	0.0239 (7)	0.0002 (5)	0.0059 (5)	0.0027 (5)
O4	0.0198 (6)	0.0245 (6)	0.0229 (6)	0.0021 (4)	0.0041 (4)	−0.0004 (5)
O5	0.0213 (6)	0.0331 (7)	0.0190 (6)	−0.0004 (5)	−0.0008 (5)	0.0014 (5)
O6	0.0203 (6)	0.0326 (7)	0.0191 (6)	−0.0027 (5)	−0.0035 (5)	0.0048 (5)
O7	0.0178 (5)	0.0327 (7)	0.0215 (6)	0.0020 (5)	0.0037 (4)	−0.0027 (5)
O8	0.0193 (6)	0.0300 (7)	0.0196 (6)	0.0003 (5)	−0.0043 (5)	−0.0006 (5)
O9	0.0235 (6)	0.0372 (7)	0.0213 (7)	0.0065 (5)	0.0007 (5)	0.0055 (5)
O10	0.0182 (5)	0.0251 (6)	0.0255 (6)	0.0033 (4)	0.0021 (5)	−0.0032 (5)
O11	0.0203 (6)	0.0371 (7)	0.0170 (6)	−0.0064 (5)	−0.0035 (5)	0.0012 (5)
O12	0.0237 (6)	0.0328 (7)	0.0183 (6)	−0.0063 (5)	0.0001 (5)	−0.0035 (5)
O13	0.0174 (5)	0.0212 (6)	0.0240 (6)	0.0016 (4)	−0.0021 (4)	0.0008 (5)
O14	0.0236 (6)	0.0413 (8)	0.0183 (6)	−0.0071 (5)	−0.0009 (5)	0.0059 (5)
O15	0.0200 (6)	0.0461 (8)	0.0170 (6)	0.0011 (5)	0.0037 (5)	0.0013 (5)
O16	0.0175 (5)	0.0266 (6)	0.0219 (6)	0.0008 (4)	−0.0040 (4)	0.0030 (5)
O17	0.0254 (6)	0.0335 (7)	0.0175 (6)	−0.0014 (5)	0.0012 (5)	−0.0017 (5)
O18	0.0210 (6)	0.0247 (6)	0.0201 (6)	−0.0005 (5)	0.0048 (5)	−0.0014 (5)
N1	0.0216 (7)	0.0167 (7)	0.0242 (8)	−0.0015 (5)	−0.0006 (5)	0.0000 (5)
N2	0.0202 (6)	0.0175 (7)	0.0190 (7)	0.0008 (5)	−0.0004 (5)	−0.0006 (5)
N3	0.0198 (6)	0.0175 (7)	0.0182 (7)	−0.0001 (5)	−0.0004 (5)	−0.0005 (5)
N4	0.0198 (6)	0.0168 (7)	0.0198 (7)	−0.0002 (5)	−0.0008 (5)	0.0020 (5)
N5	0.0200 (6)	0.0169 (7)	0.0194 (7)	0.0009 (5)	−0.0003 (5)	−0.0015 (5)
N6	0.0198 (6)	0.0178 (7)	0.0189 (7)	0.0003 (5)	0.0002 (5)	0.0019 (5)
C1	0.0309 (9)	0.0169 (8)	0.0277 (9)	−0.0044 (7)	0.0059 (7)	0.0007 (7)
C2	0.0321 (9)	0.0190 (8)	0.0300 (10)	0.0046 (7)	0.0069 (7)	0.0075 (7)
C3	0.0232 (8)	0.0228 (8)	0.0239 (9)	0.0036 (6)	0.0016 (6)	0.0039 (7)
C4	0.0190 (7)	0.0191 (8)	0.0207 (8)	−0.0009 (6)	0.0008 (6)	0.0015 (6)
C5	0.0182 (7)	0.0180 (8)	0.0160 (8)	0.0009 (6)	0.0002 (6)	0.0008 (6)
C6	0.0173 (7)	0.0165 (8)	0.0228 (9)	0.0005 (6)	−0.0021 (6)	0.0008 (6)
C7	0.0206 (8)	0.0196 (8)	0.0361 (10)	−0.0001 (6)	−0.0063 (7)	0.0015 (7)
C8	0.0233 (8)	0.0235 (8)	0.0216 (9)	0.0049 (6)	−0.0027 (6)	−0.0008 (7)
C9	0.0202 (8)	0.0213 (8)	0.0228 (9)	0.0023 (6)	0.0014 (6)	0.0007 (6)
C10	0.0304 (9)	0.0191 (8)	0.0245 (9)	0.0040 (7)	−0.0060 (7)	−0.0031 (7)
C11	0.0358 (10)	0.0173 (8)	0.0311 (10)	−0.0019 (7)	−0.0119 (8)	0.0007 (7)
C12	0.0248 (8)	0.0226 (9)	0.0275 (9)	−0.0047 (6)	−0.0047 (7)	0.0058 (7)
C13	0.0192 (7)	0.0178 (8)	0.0185 (8)	0.0010 (6)	−0.0014 (6)	0.0015 (6)
C14	0.0162 (7)	0.0215 (8)	0.0152 (8)	0.0003 (6)	−0.0027 (6)	0.0015 (6)
C15	0.0179 (7)	0.0175 (8)	0.0170 (8)	−0.0003 (6)	0.0003 (6)	0.0001 (6)
C16	0.0194 (7)	0.0189 (8)	0.0216 (9)	0.0001 (6)	0.0015 (6)	0.0001 (6)
C17	0.0218 (8)	0.0192 (8)	0.0187 (8)	0.0007 (6)	0.0005 (6)	0.0006 (6)
C18	0.0203 (8)	0.0205 (8)	0.0198 (8)	−0.0013 (6)	−0.0010 (6)	−0.0002 (6)
C19	0.0288 (9)	0.0205 (8)	0.0255 (9)	0.0026 (7)	−0.0027 (7)	−0.0013 (7)
C20	0.0354 (9)	0.0176 (8)	0.0335 (10)	−0.0045 (7)	−0.0085 (8)	0.0026 (7)
C21	0.0238 (8)	0.0246 (9)	0.0282 (10)	−0.0050 (7)	−0.0038 (7)	0.0062 (7)
C22	0.0187 (7)	0.0231 (8)	0.0175 (8)	−0.0013 (6)	−0.0006 (6)	0.0019 (6)
C23	0.0172 (7)	0.0237 (8)	0.0132 (8)	0.0003 (6)	−0.0014 (6)	0.0001 (6)
C24	0.0163 (7)	0.0174 (8)	0.0179 (8)	0.0013 (6)	0.0008 (6)	−0.0011 (6)
C25	0.0183 (7)	0.0179 (8)	0.0204 (8)	−0.0002 (6)	0.0026 (6)	0.0009 (6)
C26	0.0206 (8)	0.0210 (8)	0.0242 (9)	−0.0007 (6)	−0.0002 (6)	−0.0041 (7)
C27	0.0246 (8)	0.0215 (8)	0.0232 (9)	0.0040 (6)	0.0030 (6)	0.0012 (7)
C28	0.0292 (9)	0.0164 (8)	0.0275 (9)	0.0008 (6)	−0.0056 (7)	0.0009 (7)
C29	0.0364 (10)	0.0196 (8)	0.0309 (10)	−0.0084 (7)	−0.0070 (8)	0.0076 (7)
C30	0.0252 (8)	0.0267 (9)	0.0244 (9)	−0.0062 (7)	0.0007 (7)	0.0048 (7)
C31	0.0180 (7)	0.0217 (8)	0.0183 (8)	−0.0013 (6)	0.0002 (6)	−0.0002 (6)
C32	0.0166 (7)	0.0203 (8)	0.0142 (8)	−0.0013 (6)	−0.0005 (6)	−0.0012 (6)
C33	0.0169 (7)	0.0156 (7)	0.0180 (8)	0.0001 (6)	0.0009 (6)	0.0010 (6)
C34	0.0201 (8)	0.0183 (8)	0.0209 (8)	−0.0001 (6)	0.0036 (6)	0.0023 (6)
C35	0.0197 (7)	0.0195 (8)	0.0192 (8)	−0.0014 (6)	0.0004 (6)	0.0006 (6)
C36	0.0236 (8)	0.0195 (8)	0.0189 (8)	−0.0016 (6)	−0.0012 (6)	−0.0006 (6)
C37	0.0292 (9)	0.0178 (8)	0.0269 (9)	0.0033 (6)	0.0033 (7)	−0.0044 (7)
C38	0.0344 (9)	0.0206 (9)	0.0309 (10)	−0.0044 (7)	0.0024 (7)	−0.0109 (7)
C39	0.0228 (8)	0.0258 (9)	0.0222 (9)	−0.0041 (6)	0.0007 (6)	−0.0042 (7)
C40	0.0191 (7)	0.0195 (8)	0.0160 (8)	0.0003 (6)	0.0011 (6)	−0.0002 (6)
C41	0.0165 (7)	0.0176 (8)	0.0141 (8)	−0.0011 (6)	0.0013 (6)	0.0012 (6)
C42	0.0158 (7)	0.0166 (7)	0.0164 (8)	0.0003 (6)	−0.0013 (6)	−0.0002 (6)
C43	0.0201 (8)	0.0190 (8)	0.0240 (9)	0.0024 (6)	−0.0038 (6)	−0.0027 (6)
C44	0.0205 (8)	0.0218 (8)	0.0168 (8)	−0.0008 (6)	0.0010 (6)	−0.0011 (6)
C45	0.0178 (7)	0.0219 (8)	0.0187 (8)	−0.0012 (6)	0.0006 (6)	0.0015 (6)
C46	0.0298 (9)	0.0202 (8)	0.0228 (9)	0.0041 (7)	0.0031 (7)	0.0067 (7)
C47	0.0350 (9)	0.0182 (8)	0.0289 (10)	−0.0038 (7)	0.0079 (7)	0.0022 (7)
C48	0.0257 (8)	0.0233 (9)	0.0252 (9)	−0.0065 (6)	0.0032 (7)	−0.0016 (7)
C49	0.0183 (7)	0.0206 (8)	0.0172 (8)	−0.0001 (6)	0.0000 (6)	0.0008 (6)
C50	0.0167 (7)	0.0196 (8)	0.0142 (8)	−0.0003 (6)	0.0017 (6)	0.0002 (6)
C51	0.0157 (7)	0.0173 (7)	0.0173 (8)	0.0001 (6)	−0.0010 (6)	0.0008 (6)
C52	0.0192 (7)	0.0181 (8)	0.0215 (8)	−0.0003 (6)	−0.0028 (6)	0.0008 (6)
C53	0.0241 (8)	0.0194 (8)	0.0177 (8)	−0.0010 (6)	−0.0008 (6)	−0.0002 (6)
C54	0.0201 (7)	0.0187 (8)	0.0197 (8)	−0.0008 (6)	0.0004 (6)	0.0010 (6)

### Geometric parameters (Å, °)

**Table:**

O1—C5	1.2353 (19)	C15—C16	1.541 (2)
O2—C8	1.415 (2)	C16—H16A	0.9900
O2—H2	0.91 (3)	C16—H16B	0.9900
O3—C9	1.420 (2)	C17—H17A	0.9900
O3—H3	0.87 (3)	C17—H17B	0.9900
O4—C14	1.231 (2)	C18—H18A	0.9900
O5—C17	1.417 (2)	C18—H18B	0.9900
O5—H5	0.82 (3)	C19—C20	1.395 (3)
O6—C18	1.424 (2)	C19—H19	0.9500
O6—H6	0.90 (3)	C20—C21	1.394 (3)
O7—C23	1.2331 (19)	C20—H20	0.9500
O8—C26	1.431 (2)	C21—C22	1.394 (2)
O8—H8	0.88 (3)	C21—H21	0.9500
O9—C27	1.419 (2)	C22—C23	1.429 (2)
O9—H9	0.84 (3)	C23—C24	1.542 (2)
O10—C32	1.2307 (19)	C24—C26	1.526 (2)
O11—C35	1.434 (2)	C24—C27	1.536 (2)
O11—H11	0.85 (2)	C24—C25	1.541 (2)
O12—C36	1.4227 (19)	C25—H25A	0.9900
O12—H12	0.84 (3)	C25—H25B	0.9900
O13—C41	1.2339 (19)	C26—H26A	0.9900
O14—C44	1.419 (2)	C26—H26B	0.9900
O14—H14	0.87 (3)	C27—H27A	0.9900
O15—C45	1.422 (2)	C27—H27B	0.9900
O15—H15	0.93 (3)	C28—C29	1.394 (3)
O16—C50	1.2309 (19)	C28—H28	0.9500
O17—C53	1.420 (2)	C29—C30	1.397 (3)
O17—H17	0.88 (3)	C29—H29	0.9500
O18—C54	1.434 (2)	C30—C31	1.389 (2)
O18—H18	0.86 (2)	C30—H30	0.9500
N1—C1	1.343 (2)	C31—C32	1.434 (2)
N1—C4	1.378 (2)	C32—C33	1.536 (2)
N1—C7	1.466 (2)	C33—C36	1.526 (2)
N2—C10	1.344 (2)	C33—C35	1.526 (2)
N2—C13	1.375 (2)	C33—C34	1.545 (2)
N2—C16	1.463 (2)	C34—H34A	0.9900
N3—C19	1.343 (2)	C34—H34B	0.9900
N3—C22	1.375 (2)	C35—H35A	0.9900
N3—C25	1.462 (2)	C35—H35B	0.9900
N4—C28	1.348 (2)	C36—H36A	0.9900
N4—C31	1.376 (2)	C36—H36B	0.9900
N4—C34	1.465 (2)	C37—C38	1.395 (3)
N5—C37	1.343 (2)	C37—H37	0.9500
N5—C40	1.378 (2)	C38—C39	1.393 (3)
N5—C43	1.464 (2)	C38—H38	0.9500
N6—C46	1.342 (2)	C39—C40	1.392 (2)
N6—C49	1.377 (2)	C39—H39	0.9500
N6—C52	1.463 (2)	C40—C41	1.430 (2)
C1—C2	1.397 (3)	C41—C42	1.539 (2)
C1—H1	0.9500	C42—C44	1.528 (2)
C2—C3	1.396 (2)	C42—C45	1.531 (2)
C2—H2A	0.9500	C42—C43	1.541 (2)
C3—C4	1.394 (2)	C43—H43A	0.9900
C3—H3A	0.9500	C43—H43B	0.9900
C4—C5	1.428 (2)	C44—H44A	0.9900
C5—C6	1.534 (2)	C44—H44B	0.9900
C6—C9	1.529 (2)	C45—H45A	0.9900
C6—C8	1.537 (2)	C45—H45B	0.9900
C6—C7	1.541 (2)	C46—C47	1.394 (3)
C7—H7A	0.9900	C46—H46	0.9500
C7—H7B	0.9900	C47—C48	1.396 (3)
C8—H8A	0.9900	C47—H47	0.9500
C8—H8B	0.9900	C48—C49	1.393 (2)
C9—H9A	0.9900	C48—H48	0.9500
C9—H9B	0.9900	C49—C50	1.434 (2)
C10—C11	1.399 (3)	C50—C51	1.540 (2)
C10—H10	0.9500	C51—C54	1.528 (2)
C11—C12	1.394 (3)	C51—C53	1.534 (2)
C11—H11A	0.9500	C51—C52	1.543 (2)
C12—C13	1.395 (2)	C52—H52A	0.9900
C12—H12A	0.9500	C52—H52B	0.9900
C13—C14	1.426 (2)	C53—H53A	0.9900
C14—C15	1.542 (2)	C53—H53B	0.9900
C15—C18	1.524 (2)	C54—H54A	0.9900
C15—C17	1.536 (2)	C54—H54B	0.9900
			
C8—O2—H2	105.5 (16)	N3—C25—H25B	111.0
C9—O3—H3	113.3 (19)	C24—C25—H25B	111.0
C17—O5—H5	106.1 (17)	H25A—C25—H25B	109.0
C18—O6—H6	111.6 (16)	O8—C26—C24	108.75 (13)
C26—O8—H8	109.1 (16)	O8—C26—H26A	109.9
C27—O9—H9	109.5 (17)	C24—C26—H26A	109.9
C35—O11—H11	110.4 (16)	O8—C26—H26B	109.9
C36—O12—H12	105.8 (17)	C24—C26—H26B	109.9
C44—O14—H14	108.5 (15)	H26A—C26—H26B	108.3
C45—O15—H15	113.7 (18)	O9—C27—C24	111.68 (14)
C53—O17—H17	106.2 (17)	O9—C27—H27A	109.3
C54—O18—H18	109.3 (15)	C24—C27—H27A	109.3
C1—N1—C4	110.24 (14)	O9—C27—H27B	109.3
C1—N1—C7	135.72 (14)	C24—C27—H27B	109.3
C4—N1—C7	114.04 (13)	H27A—C27—H27B	107.9
C10—N2—C13	110.25 (14)	N4—C28—C29	107.43 (15)
C10—N2—C16	135.44 (14)	N4—C28—H28	126.3
C13—N2—C16	114.30 (13)	C29—C28—H28	126.3
C19—N3—C22	110.47 (14)	C28—C29—C30	108.09 (15)
C19—N3—C25	135.12 (14)	C28—C29—H29	126.0
C22—N3—C25	114.40 (13)	C30—C29—H29	126.0
C28—N4—C31	110.01 (14)	C31—C30—C29	106.82 (15)
C28—N4—C34	135.19 (14)	C31—C30—H30	126.6
C31—N4—C34	114.75 (13)	C29—C30—H30	126.6
C37—N5—C40	110.07 (14)	N4—C31—C30	107.64 (14)
C37—N5—C43	135.33 (14)	N4—C31—C32	108.52 (13)
C40—N5—C43	114.59 (13)	C30—C31—C32	143.79 (15)
C46—N6—C49	110.08 (14)	O10—C32—C31	128.86 (15)
C46—N6—C52	135.20 (14)	O10—C32—C33	122.74 (14)
C49—N6—C52	114.67 (13)	C31—C32—C33	108.37 (13)
N1—C1—C2	107.41 (15)	C36—C33—C35	109.85 (12)
N1—C1—H1	126.3	C36—C33—C32	109.91 (12)
C2—C1—H1	126.3	C35—C33—C32	107.49 (13)
C3—C2—C1	108.20 (15)	C36—C33—C34	112.62 (13)
C3—C2—H2A	125.9	C35—C33—C34	111.67 (13)
C1—C2—H2A	125.9	C32—C33—C34	105.07 (12)
C4—C3—C2	106.65 (15)	N4—C34—C33	103.25 (12)
C4—C3—H3A	126.7	N4—C34—H34A	111.1
C2—C3—H3A	126.7	C33—C34—H34A	111.1
N1—C4—C3	107.49 (14)	N4—C34—H34B	111.1
N1—C4—C5	108.86 (14)	C33—C34—H34B	111.1
C3—C4—C5	143.53 (15)	H34A—C34—H34B	109.1
O1—C5—C4	128.96 (14)	O11—C35—C33	107.89 (12)
O1—C5—C6	122.68 (14)	O11—C35—H35A	110.1
C4—C5—C6	108.35 (13)	C33—C35—H35A	110.1
C9—C6—C5	109.86 (13)	O11—C35—H35B	110.1
C9—C6—C8	107.51 (13)	C33—C35—H35B	110.1
C5—C6—C8	108.64 (13)	H35A—C35—H35B	108.4
C9—C6—C7	113.67 (13)	O12—C36—C33	110.70 (13)
C5—C6—C7	104.92 (13)	O12—C36—H36A	109.5
C8—C6—C7	112.15 (14)	C33—C36—H36A	109.5
N1—C7—C6	103.41 (12)	O12—C36—H36B	109.5
N1—C7—H7A	111.1	C33—C36—H36B	109.5
C6—C7—H7A	111.1	H36A—C36—H36B	108.1
N1—C7—H7B	111.1	N5—C37—C38	107.29 (15)
C6—C7—H7B	111.1	N5—C37—H37	126.4
H7A—C7—H7B	109.0	C38—C37—H37	126.4
O2—C8—C6	111.33 (14)	C39—C38—C37	108.50 (15)
O2—C8—H8A	109.4	C39—C38—H38	125.8
C6—C8—H8A	109.4	C37—C38—H38	125.8
O2—C8—H8B	109.4	C40—C39—C38	106.47 (15)
C6—C8—H8B	109.4	C40—C39—H39	126.8
H8A—C8—H8B	108.0	C38—C39—H39	126.8
O3—C9—C6	112.89 (13)	N5—C40—C39	107.67 (14)
O3—C9—H9A	109.0	N5—C40—C41	108.64 (13)
C6—C9—H9A	109.0	C39—C40—C41	143.69 (15)
O3—C9—H9B	109.0	O13—C41—C40	128.95 (14)
C6—C9—H9B	109.0	O13—C41—C42	122.72 (14)
H9A—C9—H9B	107.8	C40—C41—C42	108.32 (13)
N2—C10—C11	107.45 (15)	C44—C42—C45	108.87 (13)
N2—C10—H10	126.3	C44—C42—C41	109.46 (12)
C11—C10—H10	126.3	C45—C42—C41	107.85 (12)
C12—C11—C10	107.97 (15)	C44—C42—C43	112.88 (13)
C12—C11—H11A	126.0	C45—C42—C43	112.57 (13)
C10—C11—H11A	126.0	C41—C42—C43	105.02 (12)
C11—C12—C13	106.87 (15)	N5—C43—C42	103.40 (12)
C11—C12—H12A	126.6	N5—C43—H43A	111.1
C13—C12—H12A	126.6	C42—C43—H43A	111.1
N2—C13—C12	107.45 (14)	N5—C43—H43B	111.1
N2—C13—C14	109.24 (13)	C42—C43—H43B	111.1
C12—C13—C14	143.30 (16)	H43A—C43—H43B	109.0
O4—C14—C13	129.15 (15)	O14—C44—C42	111.44 (13)
O4—C14—C15	122.96 (14)	O14—C44—H44A	109.3
C13—C14—C15	107.88 (13)	C42—C44—H44A	109.3
C18—C15—C17	108.93 (13)	O14—C44—H44B	109.3
C18—C15—C16	112.08 (13)	C42—C44—H44B	109.3
C17—C15—C16	113.26 (13)	H44A—C44—H44B	108.0
C18—C15—C14	108.44 (13)	O15—C45—C42	110.90 (13)
C17—C15—C14	108.88 (12)	O15—C45—H45A	109.5
C16—C15—C14	105.06 (12)	C42—C45—H45A	109.5
N2—C16—C15	103.49 (12)	O15—C45—H45B	109.5
N2—C16—H16A	111.1	C42—C45—H45B	109.5
C15—C16—H16A	111.1	H45A—C45—H45B	108.0
N2—C16—H16B	111.1	N6—C46—C47	107.72 (15)
C15—C16—H16B	111.1	N6—C46—H46	126.1
H16A—C16—H16B	109.0	C47—C46—H46	126.1
O5—C17—C15	111.92 (13)	C46—C47—C48	107.93 (15)
O5—C17—H17A	109.2	C46—C47—H47	126.0
C15—C17—H17A	109.2	C48—C47—H47	126.0
O5—C17—H17B	109.2	C49—C48—C47	106.85 (15)
C15—C17—H17B	109.2	C49—C48—H48	126.6
H17A—C17—H17B	107.9	C47—C48—H48	126.6
O6—C18—C15	108.77 (13)	N6—C49—C48	107.42 (14)
O6—C18—H18A	109.9	N6—C49—C50	108.56 (13)
C15—C18—H18A	109.9	C48—C49—C50	144.01 (15)
O6—C18—H18B	109.9	O16—C50—C49	129.06 (14)
C15—C18—H18B	109.9	O16—C50—C51	122.65 (14)
H18A—C18—H18B	108.3	C49—C50—C51	108.30 (13)
N3—C19—C20	107.30 (15)	C54—C51—C53	108.44 (13)
N3—C19—H19	126.3	C54—C51—C50	108.67 (13)
C20—C19—H19	126.3	C53—C51—C50	109.45 (12)
C21—C20—C19	108.12 (15)	C54—C51—C52	112.50 (13)
C21—C20—H20	125.9	C53—C51—C52	112.71 (13)
C19—C20—H20	125.9	C50—C51—C52	104.93 (12)
C22—C21—C20	106.90 (15)	N6—C52—C51	103.51 (12)
C22—C21—H21	126.6	N6—C52—H52A	111.1
C20—C21—H21	126.6	C51—C52—H52A	111.1
N3—C22—C21	107.20 (15)	N6—C52—H52B	111.1
N3—C22—C23	108.97 (13)	C51—C52—H52B	111.1
C21—C22—C23	143.82 (16)	H52A—C52—H52B	109.0
O7—C23—C22	129.30 (15)	O17—C53—C51	111.41 (13)
O7—C23—C24	122.61 (14)	O17—C53—H53A	109.3
C22—C23—C24	108.09 (13)	C51—C53—H53A	109.3
C26—C24—C27	109.57 (13)	O17—C53—H53B	109.3
C26—C24—C25	112.05 (13)	C51—C53—H53B	109.3
C27—C24—C25	112.37 (13)	H53A—C53—H53B	108.0
C26—C24—C23	107.96 (13)	O18—C54—C51	109.04 (13)
C27—C24—C23	109.81 (12)	O18—C54—H54A	109.9
C25—C24—C23	104.88 (12)	C51—C54—H54A	109.9
N3—C25—C24	103.62 (12)	O18—C54—H54B	109.9
N3—C25—H25A	111.0	C51—C54—H54B	109.9
C24—C25—H25A	111.0	H54A—C54—H54B	108.3
			
C4—N1—C1—C2	0.14 (19)	C31—N4—C28—C29	−0.26 (19)
C7—N1—C1—C2	179.88 (18)	C34—N4—C28—C29	−177.52 (17)
N1—C1—C2—C3	−0.5 (2)	N4—C28—C29—C30	0.1 (2)
C1—C2—C3—C4	0.7 (2)	C28—C29—C30—C31	0.2 (2)
C1—N1—C4—C3	0.30 (19)	C28—N4—C31—C30	0.36 (19)
C7—N1—C4—C3	−179.50 (14)	C34—N4—C31—C30	178.23 (14)
C1—N1—C4—C5	−176.64 (14)	C28—N4—C31—C32	−177.71 (14)
C7—N1—C4—C5	3.56 (19)	C34—N4—C31—C32	0.16 (19)
C2—C3—C4—N1	−0.62 (19)	C29—C30—C31—N4	−0.31 (19)
C2—C3—C4—C5	174.5 (2)	C29—C30—C31—C32	176.6 (2)
N1—C4—C5—O1	179.58 (16)	N4—C31—C32—O10	179.08 (16)
C3—C4—C5—O1	4.5 (4)	C30—C31—C32—O10	2.2 (4)
N1—C4—C5—C6	0.81 (18)	N4—C31—C32—C33	1.16 (17)
C3—C4—C5—C6	−174.3 (2)	C30—C31—C32—C33	−175.7 (2)
O1—C5—C6—C9	54.1 (2)	O10—C32—C33—C36	58.6 (2)
C4—C5—C6—C9	−127.00 (14)	C31—C32—C33—C36	−123.34 (14)
O1—C5—C6—C8	−63.2 (2)	O10—C32—C33—C35	−60.95 (19)
C4—C5—C6—C8	115.65 (15)	C31—C32—C33—C35	117.13 (14)
O1—C5—C6—C7	176.68 (15)	O10—C32—C33—C34	179.98 (14)
C4—C5—C6—C7	−4.45 (17)	C31—C32—C33—C34	−1.93 (17)
C1—N1—C7—C6	174.07 (18)	C28—N4—C34—C33	175.80 (17)
C4—N1—C7—C6	−6.20 (19)	C31—N4—C34—C33	−1.37 (18)
C9—C6—C7—N1	126.15 (14)	C36—C33—C34—N4	121.53 (14)
C5—C6—C7—N1	6.10 (17)	C35—C33—C34—N4	−114.32 (14)
C8—C6—C7—N1	−111.65 (15)	C32—C33—C34—N4	1.91 (16)
C9—C6—C8—O2	−178.12 (13)	C36—C33—C35—O11	−168.99 (12)
C5—C6—C8—O2	−59.28 (17)	C32—C33—C35—O11	−49.42 (16)
C7—C6—C8—O2	56.23 (18)	C34—C33—C35—O11	65.33 (16)
C5—C6—C9—O3	50.03 (17)	C35—C33—C36—O12	179.10 (12)
C8—C6—C9—O3	168.08 (13)	C32—C33—C36—O12	61.02 (17)
C7—C6—C9—O3	−67.18 (18)	C34—C33—C36—O12	−55.75 (17)
C13—N2—C10—C11	1.16 (19)	C40—N5—C37—C38	−0.29 (19)
C16—N2—C10—C11	−179.96 (17)	C43—N5—C37—C38	−179.10 (17)
N2—C10—C11—C12	−1.0 (2)	N5—C37—C38—C39	0.0 (2)
C10—C11—C12—C13	0.4 (2)	C37—C38—C39—C40	0.2 (2)
C10—N2—C13—C12	−0.91 (19)	C37—N5—C40—C39	0.44 (19)
C16—N2—C13—C12	179.94 (14)	C43—N5—C40—C39	179.52 (14)
C10—N2—C13—C14	178.12 (14)	C37—N5—C40—C41	−179.23 (14)
C16—N2—C13—C14	−1.02 (19)	C43—N5—C40—C41	−0.15 (19)
C11—C12—C13—N2	0.29 (19)	C38—C39—C40—N5	−0.41 (19)
C11—C12—C13—C14	−178.2 (2)	C38—C39—C40—C41	179.1 (2)
N2—C13—C14—O4	−178.10 (16)	N5—C40—C41—O13	−179.63 (15)
C12—C13—C14—O4	0.4 (4)	C39—C40—C41—O13	0.9 (4)
N2—C13—C14—C15	1.49 (17)	N5—C40—C41—C42	−1.05 (17)
C12—C13—C14—C15	179.9 (2)	C39—C40—C41—C42	179.5 (2)
O4—C14—C15—C18	58.23 (19)	O13—C41—C42—C44	−58.11 (19)
C13—C14—C15—C18	−121.39 (14)	C40—C41—C42—C44	123.21 (14)
O4—C14—C15—C17	−60.2 (2)	O13—C41—C42—C45	60.19 (19)
C13—C14—C15—C17	120.22 (14)	C40—C41—C42—C45	−118.50 (14)
O4—C14—C15—C16	178.23 (14)	O13—C41—C42—C43	−179.55 (15)
C13—C14—C15—C16	−1.39 (17)	C40—C41—C42—C43	1.76 (16)
C10—N2—C16—C15	−178.75 (17)	C37—N5—C43—C42	−179.98 (18)
C13—N2—C16—C15	0.10 (18)	C40—N5—C43—C42	1.25 (18)
C18—C15—C16—N2	118.34 (14)	C44—C42—C43—N5	−120.92 (14)
C17—C15—C16—N2	−117.93 (14)	C45—C42—C43—N5	115.33 (14)
C14—C15—C16—N2	0.78 (16)	C41—C42—C43—N5	−1.74 (16)
C18—C15—C17—O5	−176.35 (13)	C45—C42—C44—O14	−175.90 (12)
C16—C15—C17—O5	58.21 (17)	C41—C42—C44—O14	−58.24 (17)
C14—C15—C17—O5	−58.27 (17)	C43—C42—C44—O14	58.33 (17)
C17—C15—C18—O6	166.48 (12)	C44—C42—C45—O15	168.90 (12)
C16—C15—C18—O6	−67.40 (16)	C41—C42—C45—O15	50.23 (16)
C14—C15—C18—O6	48.12 (16)	C43—C42—C45—O15	−65.15 (16)
C22—N3—C19—C20	−0.12 (19)	C49—N6—C46—C47	0.03 (19)
C25—N3—C19—C20	−179.88 (17)	C52—N6—C46—C47	177.21 (17)
N3—C19—C20—C21	0.3 (2)	N6—C46—C47—C48	0.1 (2)
C19—C20—C21—C22	−0.4 (2)	C46—C47—C48—C49	−0.2 (2)
C19—N3—C22—C21	−0.14 (19)	C46—N6—C49—C48	−0.13 (19)
C25—N3—C22—C21	179.66 (14)	C52—N6—C49—C48	−177.95 (14)
C19—N3—C22—C23	−179.59 (14)	C46—N6—C49—C50	179.19 (13)
C25—N3—C22—C23	0.22 (19)	C52—N6—C49—C50	1.38 (18)
C20—C21—C22—N3	0.35 (19)	C47—C48—C49—N6	0.18 (19)
C20—C21—C22—C23	179.5 (2)	C47—C48—C49—C50	−178.7 (2)
N3—C22—C23—O7	−179.57 (16)	N6—C49—C50—O16	178.45 (16)
C21—C22—C23—O7	1.3 (4)	C48—C49—C50—O16	−2.6 (4)
N3—C22—C23—C24	1.09 (17)	N6—C49—C50—C51	−1.64 (17)
C21—C22—C23—C24	−178.0 (2)	C48—C49—C50—C51	177.3 (2)
O7—C23—C24—C26	−61.64 (19)	O16—C50—C51—C54	60.67 (19)
C22—C23—C24—C26	117.76 (14)	C49—C50—C51—C54	−119.24 (14)
O7—C23—C24—C27	57.8 (2)	O16—C50—C51—C53	−57.58 (19)
C22—C23—C24—C27	−122.83 (14)	C49—C50—C51—C53	122.50 (14)
O7—C23—C24—C25	178.73 (14)	O16—C50—C51—C52	−178.78 (14)
C22—C23—C24—C25	−1.88 (17)	C49—C50—C51—C52	1.31 (16)
C19—N3—C25—C24	178.35 (17)	C46—N6—C52—C51	−177.59 (17)
C22—N3—C25—C24	−1.40 (17)	C49—N6—C52—C51	−0.50 (18)
C26—C24—C25—N3	−114.97 (14)	C54—C51—C52—N6	117.47 (14)
C27—C24—C25—N3	121.14 (14)	C53—C51—C52—N6	−119.54 (14)
C23—C24—C25—N3	1.90 (16)	C50—C51—C52—N6	−0.51 (16)
C27—C24—C26—O8	−168.30 (12)	C54—C51—C53—O17	179.10 (13)
C25—C24—C26—O8	66.26 (16)	C50—C51—C53—O17	−62.50 (17)
C23—C24—C26—O8	−48.74 (16)	C52—C51—C53—O17	53.87 (17)
C26—C24—C27—O9	176.49 (13)	C53—C51—C54—O18	167.38 (12)
C25—C24—C27—O9	−58.26 (17)	C50—C51—C54—O18	48.49 (16)
C23—C24—C27—O9	58.07 (17)	C52—C51—C54—O18	−67.26 (16)

### Hydrogen-bond geometry (Å, °)

**Table:**

*D*—H···*A*	*D*—H	H···*A*	*D*···*A*	*D*—H···*A*
O2—H2···O3^i^	0.91 (3)	1.85 (3)	2.7513 (19)	172 (2)
O3—H3···O4	0.87 (3)	1.95 (3)	2.7612 (17)	154 (3)
O5—H5···O6^ii^	0.82 (3)	1.98 (3)	2.7776 (17)	166 (2)
O6—H6···O1^iii^	0.90 (3)	1.80 (3)	2.6801 (16)	168 (2)
O8—H8···O16	0.88 (3)	1.85 (3)	2.7219 (16)	168 (2)
O9—H9···O11^iv^	0.84 (3)	1.96 (3)	2.7841 (18)	168 (2)
O11—H11···O13^v^	0.85 (2)	1.92 (2)	2.7239 (16)	157 (2)
O12—H12···O8	0.84 (3)	2.00 (3)	2.8186 (18)	167 (2)
O14—H14···O18	0.87 (3)	1.95 (3)	2.8063 (18)	168 (2)
O15—H15···O10^vi^	0.93 (3)	1.86 (3)	2.7290 (16)	155 (3)
O17—H17···O15^v^	0.88 (3)	1.96 (3)	2.8144 (18)	166 (3)
O18—H18···O7^vii^	0.86 (2)	1.92 (2)	2.7579 (17)	168 (2)

Symmetry codes: (i) *x*, −*y*+1/2, *z*+1/2; (ii) *x*, −*y*+1/2, *z*−1/2; (iii) *x*+1, *y*, *z*; (iv) *x*, *y*, *z*+1; (v) *x*, *y*, *z*−1; (vi) *x*−1, *y*, *z*+1; (vii) *x*−1, *y*, *z*.
